# Functional outcome after Brachytherapy with bi-nuclide (Ru-106/Iodine-125) plaques in large uveal melanomas

**DOI:** 10.1186/s13014-024-02576-6

**Published:** 2025-02-12

**Authors:** Leyla Jabbarli, Miltiadis Fiorentzis, Philipp Rating, Boerge Schmidt, Eva Biewald, Nika Guberina, Dirk Flühs, Norbert Bornfeld, Wolfgang Sauerwein, Martin Stuschke, Nikolaos E. Bechrakis, Maja Guberina

**Affiliations:** 1 Department of Ophthalmology, University Hospital Essen, National Center for Tumor Diseases (NCT) West, D-45147 Essen, Germany; 2 Institute for Medical Informatics, Biometry and Epidemiology (IMIBE), University of Duisburg-Essen, Essen, Germany; 3 Department of Radiotherapy, University Hospital Essen, National Center for Tumor Diseases (NCT) West, Essen, Germany; 4 German Cancer Consortium (DKTK), Partner Site University Hospital Essen, Essen, Germany

**Keywords:** Uveal melanoma, Brachytherapy, Bi-nuclide plaque, ^106^Ruthenium, ^125^Iodine, Visual acuity

## Abstract

**Objective:**

Preservation of visual acuity remains a challenging issue after globe sparing therapy of large uveal melanoma. The aim of our study was analyzing the functional outcome after brachytherapy with bi-nuclide plaques (BBNP), maintaining prognostic factors for legal blindness (LB).

**Methods:**

We have analyzed all consecutive patients with large uveal melanoma treated with BBNP at our institution between 01/1999 and 12/2020. The post-treatment follow-up data were screened up to 06/2023. Univariate and multivariate Cox regression analysis was performed to identify predictive factors for development of LB following BBNP.

**Results:**

Overall, 570 patients with median age of 65.6 years (interquartile range [IQR]: 54.5–74.0) underwent BBNP. During the median post-treatment follow-up of 30.8 months (IQR: 12.9–57.3), LB was diagnosed in 287 (50.4%) patients. Patients’ age (> 67 years, adjusted hazard ratio [aHR] = 1.58, 95%-confidence interval [CI] = 1.24–2.00, *p* < 0.0001), tumor thickness (> 8.5 mm, aHR = 1.43, 95%-CI = 1.12–1.82, *p* = 0.004), VA (> 0.5 LogMAR, aHR = 1.59, 95%-CI = 1.25–2.02, *p* < 0.0001), and ciliary body involvement (aHR = 0.77, 95%-CI = 0.60–0.97, *p* = 0.029) were confirmed as independent predictors of LB in the final multivariable Cox regression analysis.

**Conclusions:**

Approximately a half of patients with large uveal melanoma develop LB around 2.5 years after brachytherapy. Further optimization of treatment strategies, including both therapeutic and preventive measures, has the potential to enhance the functional outcome after episcleral plaque therapy for large UMs.

## Introduction

With an annual incidence of six per million in Caucasians, uveal melanoma (UM) is the most common intraocular tumor in adults [1, 2]. With continuous improvement of treatment strategies, a globe sparing therapy with preserving visual acuity (VA) is feasible even in large tumors [3–6]

Visual outcome after brachytherapy for UM is difficult to predict, considering that it is a result of many factors. Some of this factors are not modifiable such as tumor location in relation to fovea and optic nerve or tumor thickness. Other factors are therapy associated, such as radiation retinopathy (RR), radiation maculopathy (RM), and radiation opticopathy (RO) [7]. The incidence of these complications is related to applied radiation dose to radiosensitive structures, such as macula or optic nerve as well as the size of the irradiated volume [8]. The Collaborative Ocular Melanoma Study (COMS) reported a significant visual loss by almost 50% of the patients three years after brachytherapy with iodine-125 (^125^I) with a prescribed dose of 85 Gy to a minimum of 5 mm from the inner sclera or to the tumor apex if greater than 5 mm [9]. There are several studies demonstrating better visual outcome with decreased applied radiation doses with ^125^I plaques and after brachytherapy with the beta-emitter ruthenium-106 (^106^Ru), which’s irradiated volume is considerably smaller as compared to the irradiated volume of an iodine plaque [10–13]. Therefore, radiation dose and irradiated volume seem to be essential modifiable risk factors for the preservation of visual outcome after treatment [14]. Overall, choosing the optimal radiation dose is a challenge, considering that the adjacent structures must be protected from unnecessary radiation exposure and that the highest possible radiation doses are required to successfully treat large eye tumors [15]. 

In order to reduce the collateral damage to healthy ocular tissues, preserve functionality of the eye as much as possible with concurrently high local tumor control, we use since 1997 at our institution bi-nuclide radioactive plaques (BBNP) with ^106^Ru and ^125^I. Due to steeper dose gradient of ^106^Ru at a distance of 5 mm from the plaque surface, the beta particles are almost completely absorbed, and just minimal photon exposure can be detected, which means a considerable reduction of applied dose and sparing of radiosensitive structures [16]. A combination with ^125^I with ^106^Ru enables an effective irradiation of tumors > 7 mm with high local tumor control. However, no published data currently exists on the safety and efficacy of such treatment of large UM with BBNP with regard to the preservation of the visual outcome.

Therefore, we aimed at analyzing the functional outcome after BBNP for large UM in a retrospective observational cohort study covering the treatment period of over 20 years in a large tertiary university hospital in Germany. The special emphasis was put on the identification of risk factors related the deterioration of VA and development of LB in the postoperative course.

## Methods and materials

### Patient population

We have reviewed clinical records of all patients with UM treated at the Department of Ophthalmology of the University Hospital Essen between 01/1999 and 12/2020. The patients with large UM (tumor thickness ≥ 7 mm) managed with BBNP were included in our study. The patients without VA data at diagnosis and those managed initially with two plaques (bi-nuclide and ^106^Ru) were excluded. This study was conducted in accordance with the Declaration of Helsinki and was registered in the German clinical trial registry (DRKS, Unique identifier: DRKS00019049, registration date 10.21.2019). The approval of the Institutional Ethics Committee (Medical faculty of the University Duisburg-Essen, registration number 20-9165-BO) was obtained. All patients signed the informed consent within the written treatment contract on admission.

### Clinical management of UM

A detailed ophthalmologic examination was performed initially and at each follow-up visit after brachytherapy. A tumor documentation was performed with colored fundus photos, ultrasonography or/and ultrasound biomicroscopy. UM was mostly diagnosed clinically. A tumor thickness of > 7 mm was the indication for BBNP. The patients with extensive exudative retinal detachment became intravitreal triamcinolone at a dose of 4 mg (0.1 ml) using a 30-gauge needle with trans pars plana approach following directly plaque suturing or plaque removal surgery.

**Fig. 1:**
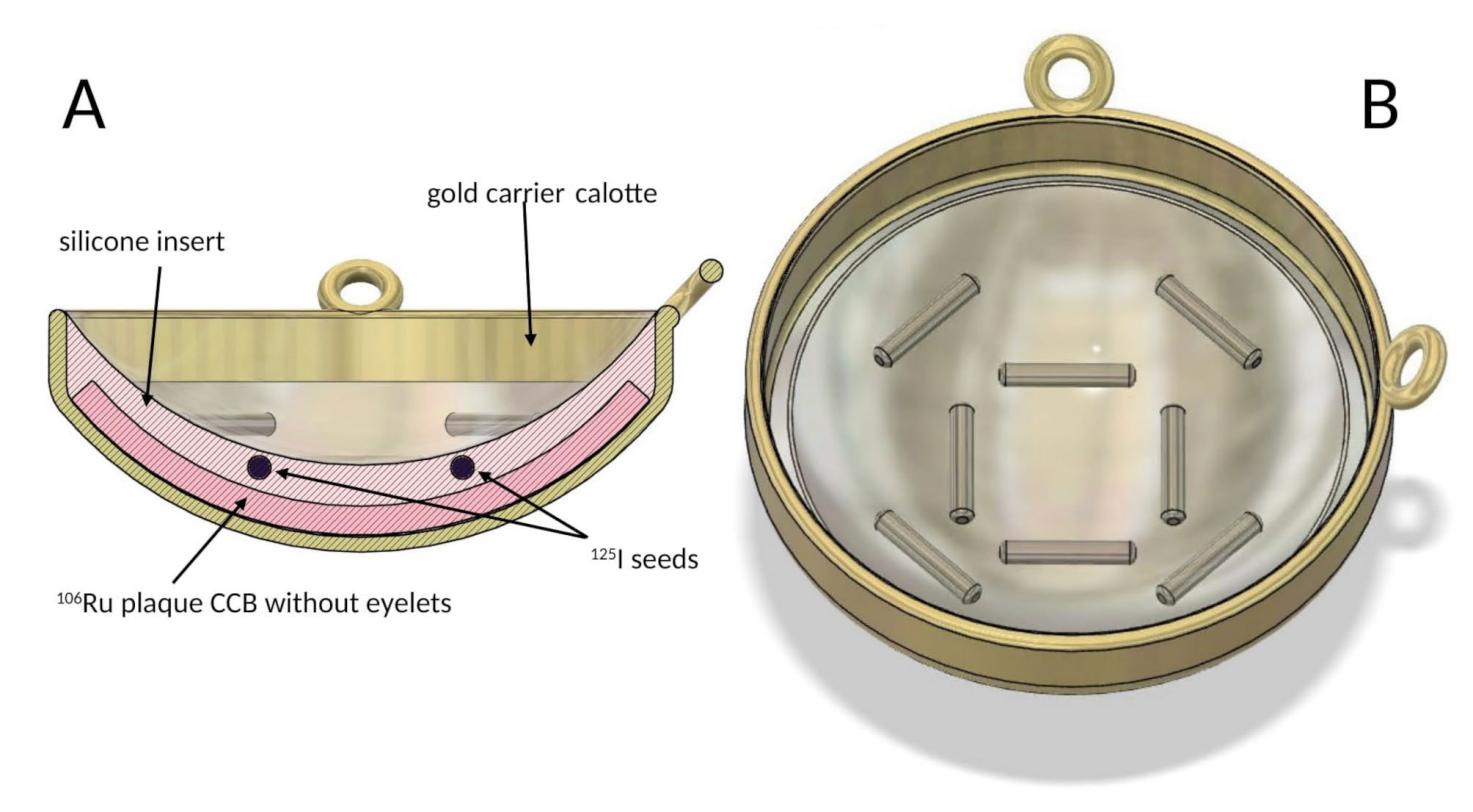
Design of a bi-nuclide plaque. **A** - Bi-nuclide plaque in cross section. **B** - Inner surface of bi-nuclide plaque with radiation sources

A detailed design description of bi-nuclide radioactive plaques was published previously [16]. In short, a bi-nuclide plaque consisted of a gold calotte with two fixation eyelets for suturing the plaque onto the eye (designed by W. Sauerwein and D. Flühs and manufactured by Schmuck Merath, Ulm, Germany), a dedicated 20 mm ^106^Ru plaque without eyelets (CCB type manufactured by BEBIG, Berlin, Germany) and 8–12 ^125^I seeds (Amersham type 6711) in silicone inset (Fig. 1). The prescribed minimum dose for the tumor tip was 120 Gy and 100 Gy in cases with tumor height of < 8 mm and > 8 mm respectively, whereby the sclera dose was at least 700 Gy but did not exceed 1500 Gy [17].

After brachytherapy, the follow-up intervals were every 3-months in the first year. In absence of complications, the intervals were prolonged up to once every year.

### Data management

Apart of basic demographic patient characteristics (age and sex), the following tumor and radiation data were extracted: largest tumor thickness and largest basal tumor diameter prior to the therapy (based on ultrasonography), location of posterior tumor margin (peripapillary, posterior to equator, anterior to equator), ciliary body involvement, extraocular tumor extension, radiation dose to tumor apex and sclera, radiation duration. The cases with posterior tumor margin within 5 mm proximity to optic nerve were defined as peripapillary tumors.

The extracted clinical data consisted of: date of brachytherapy, the last documented follow-up, VA at diagnosis and at the last follow up. Therapy associated documented variables were scleral necrosis, RM, RR, treatment with laser photocoagulation, intravitreal or subtenonal therapy with vascular endothelial growth factor (VEGF) inhibitors and/or triamcinolone. Radiation induced retinal vessel pathology clinically presenting with hemorrhages, microaneurysms, nerve fibre layer infarctions, retinal exudation, with or without neovascularization in peripheral retina, was defined as RR. Radiation induced macular pathology manifesting as macular edema, hemorrhages, hard exudations or macular atrophy was defined as RM.

VA was measured on decimal scale chart at a distance of 5 m ranging from 1.0 to 0.05 decimal. A VA from 0.04 to 0.02 decimal was measured with VA board at a distance of 1 m. A VA worse than 0.02 decimal was recorded as counting fingers at 1 m, hand movement, light perception, and no light perception. VA was classified based on the International Classification of Diseases (ICD) 11 (2019) distance visual impairment classification [18] as mild or no visual impairment (VI) (VA ≥ 0.3 decimal), moderate VI (< 0.3 to ≥ 0.1 decimal), severe VI (< 0.1 to ≥ 0.05 decimal) and LB (< 0.05 decimal). For further statistical assessment, VA data were converted to LogMAR units (logarithm of the Minimum Angle Resolution). For the assessment of VA and all above-mentioned post-treatment complications, patients’ electronic health records were screened up to June 2023.

### Study endpoints and statistical analysis

The primary endpoint of the study was the analysis of visual outcome after BBNP with the identification of prognostic factors for legal blindness (LB) defined as a VA of > 1.3 LogMAR. Data analysis was performed with the use of SPSS (version 25, SPSS Inc., IBM, Chicago, IL, USA). A *p*-value of 0.05 or less were considered as significant. For descriptive data, absolute numbers (with percentages) were used for categorical variables, whereas the continuous variables were reported using median values and interquartile range [IQR].

The associations between the baseline characteristics and occurrence of LB were analyzed in univariate and multivariate Cox regression models. Prior to inclusion to univariate analysis, continuous variables were dichotomized according to the cutoffs identified in the receiver operating characteristic (ROC) curves. The significant parameters from the univariable analysis were then included in the final multivariable Cox regression analysis to reveal the independent prognostic factors for LB. Kaplan–Meier survival analysis were performed to show the cumulative effect of the significant predictors on LB occurrence.

## Results

### Description of patient population

Between 01/1999 and 12/2020, 594 patients with large UM undergone BBNP at the Department of Ophthalmology and Radiotherapy of the University Hospital Essen. Eighteen patients were managed with two plaques (bi-nuclide and ^106^Ru) as primary therapy and were therefore excluded from this study. Six patients were excluded from final analysis due to missing initial VA value. Therefore, the median age of 570 individuals included in the final analysis was 65.6 years (IQR: 54.5–74.0), 276 (48.4%) patients were female. Table 1 demonstrates the major baseline patients, tumor and treatment features.

**Table 1:** Major baseline patients, tumor and treatment features

Parameter	Number of cases (%) or median value (IQR)
Age, years	65.6 (54.5–74.0)
Sex, female	276 (48.4%)
TNM category	
T2a T2b T3a	35 (6.1%) 39 (6.8%) 203 (35.6%)
T3b T3c	225 (39.5%) 6 (1.1%)
T3d T4a	17 (3.0%) 15 (2.6%)
T4b T4c	28 (4.9%) 2 (0.4%)
Tumor thickness prior the therapy, mm	8.6 (7.9–9.6)
Largest basal tumor diameter, mm	15.0 (13.3–16.4)
Posterior tumor margin*:	
Peripapillary Anterior to equator Posterior to equator	153 (36.1%) 137 (32.4%) 132 (31.3%)
Extraocular extension	28 (4.9%)
Ciliary body involvement	309 (54.2%)
Iris involvement	0 (0%)
Radiation induced scleral necrosis	68 (11.9%)
Visual acuity at diagnosis, LogMAR	0.4 (0.1–0.7)
Visual acuity at last follow-up, LogMAR	1.4 (1.0-2.3)
Radiation retinopathy	163 (28.6%)
Radiation maculopathy	189 (33.2%)
Intravitreal therapy with Anti-VEGF or triamcinolone	55 (9.6%)
Triamcinolone intravitreal during brachytherapy	190 (33.3%)
Transpupillary thermotherapy	23 (4.0%)
Apex dose, Gy	74.5 (70.5–86.8)
Sclera dose, Gy	937.4 (812.9–1108.5)
Radiation duration, hour	140.0 (101.4–184.8)
Follow up duration, months	30.8 (12.9–57.3)

**Abbreviations**: IQR – interquartile range; TNM-tumor, node, metastasis; Anti- VEGF- Anti–vascular endothelial growth factor; *- No available information in 146 patients

The median initial VA at diagnosis was 0.4 LogMAR (IQR: 0.1–0.7). According to the ICD-11 (2019) VI classification, the following VA was documented at diagnosis of UM: Mild or no VI (VA ≤ 0.52 LogMAR) in 380 (66.0%) patients, moderate VI (VA > 0.52 to 1.0 LogMAR) in 128 (22. %) cases, severe VI (VA > 1.0 to ≤ 1.3 LogMAR) in 22 (3. %) cases, and LB (> 1.3 LogMAR, ) in 40 (6. %) cases.

**Fig. 2:**
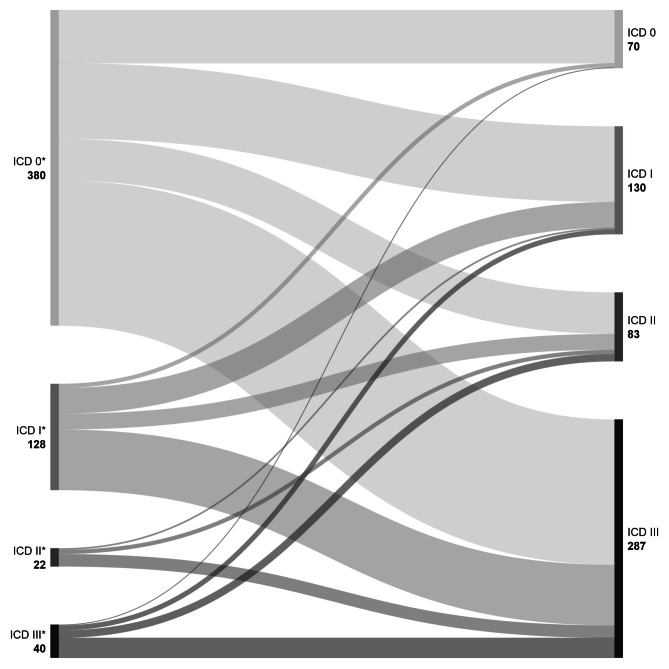
The change in VA during the whole documented observational period, based on the ICD-11 visual impairment classification

Of 508 (88.2%) patients with VA ≤ 1.0 LogMAR at initial presentation, only 190 (33.0%) individuals preserved this VA after the median follow up of 30.8 months (IQR: (12.9–57.4) months). The median final VA was 1.4 LogMAR (IQR: 1.0–2.3). Altogether, 70 (12.3%) patients showed mild or no VI at last visit. Moderate and severe VI was recorded in 129 (22.8%) and 83 (14.6%) cases, respectively. LB at last visit was documented in 287 (50.4%) patients. The change in VA based on the ICD-11 VI classification, during the whole documented observational period is shown in the appropriate Sankey diagram (Fig. 2).

RR was diagnosed in 28.6% (*n* = 163) cases. 189 (33.2%) patients developed RM.

The univariable Cox regression analysis revealed the following parameters as significant risk factors for development of LB in the affected eyes: patients’ age > 67 years (aHR = 1.59; *p* < 0.0001), tumor thickness > 8.5 mm (aHR = 1.32; *p* = 0.0019), VA > 0.5 LogMAR (aHR = 1.55; *p* < 0.0001), ciliary body involvement (aHR = 0.78; *p* = 0.038), and radiation duration > 130 h (aHR = 0.75; *p* = 0.026) (s. Table 2).

**Table 2:** Univariable Cox regression analysis of the predictors of development of blindness after brachytherapy with bi-nuclide plagues of large uveal melanoma (tumor thickness ≥ 7 mm)

Parameter	HR (95%-CI)	*p*-value
Age > 67 years	1.59 (1.25–2.02)	**< 0.0001**
Sex, female	1.08 (0.86–1.36)	0.519
TNM, T4 vs. T3 or T2	1.08 (0.68–1.72)	0.754
Tumor thickness > 8.5 mm	1.32 (1.05–1.67)	**0.0019**
Largest basal tumor diameter > 15 mm	1.00 (0.95–1.05)	0.991
Posterior tumor margin:		
Peripapillary vs. any other location Posterior to equator vs. any other location	1.06 (0.79–1.40) 1.19 (0.89–1.60)	0.715 0.247
Anterior to equator vs. any other location	0.77 (0.56–1.06)	0.106
Extraocular extension	1.19 (0.67–2.13)	0.550
Ciliary body involvement	0.78 (0.62–0.99)	**0.038**
Visual acuity at diagnosis > 0.5 LogMAR	1.55 (1.22–1.97)	**< 0.0001**
Adjuvant transpupillary thermotherapy	0.87 (0.52–1.46)	0.591
Apex dose, > 75 Gy	1.07 (0.84–1.35)	0.603
Sclera dose > 1000 Gy	1.02 (0.80–1.30)	0.893
Radiation duration, > 130 h*	0.75 (0.58–0.97)	**0.026**

**Abbreviation**: LogMAR-Logarithm of the Minimum Angle of Resolution; HR- Hazard ratio; CI- Confidence interval; TNM- tumor, node, metastasis;

The final multivariable Cox regression analysis confirmed patients’ age > 67 years (aHR = 1.58, *p* < 0.0001), tumor thickness (> 8.5 mm, aHR = 1.43, *p* = 0.004), VA (> 0.5 LogMAR, aHR = 1.59, *p* < 0.0001), and ciliary body involvement (aHR = 0.77, *p* = 0.029) as independent predictors for development of LB (Table 3).

**Table 3:** Multivariable Cox regression analysis of predictors for legal blindness after brachytherapy with bi-nuclide plagues of large uveal melanoma (tumor thickness ≥ 7 mm)

Parameter	aHR (95%-CI)	*p*-value
Age > 67 years	1.58 (1.24-2.00)	**< 0.0001**
Tumor thickness > 8.5 mm	1.43 (1.12–1.82)	**0.004**
Visual acuity at diagnosis > 0.5 logMAR	1.59 (1.25–2.02)	**< 0.0001**
Radiation duration > 130 h	0.81 (0.53–1.23)	0.287
Ciliary body involvement	0.77 (0.60–0.97)	**0.029**

**Abbreviations**: LogMAR-Logarithm of the Minimum Angle of Resolution; aHR-adjusted hazard ratio; CI- Confidence interval;

**Fig. 3:**
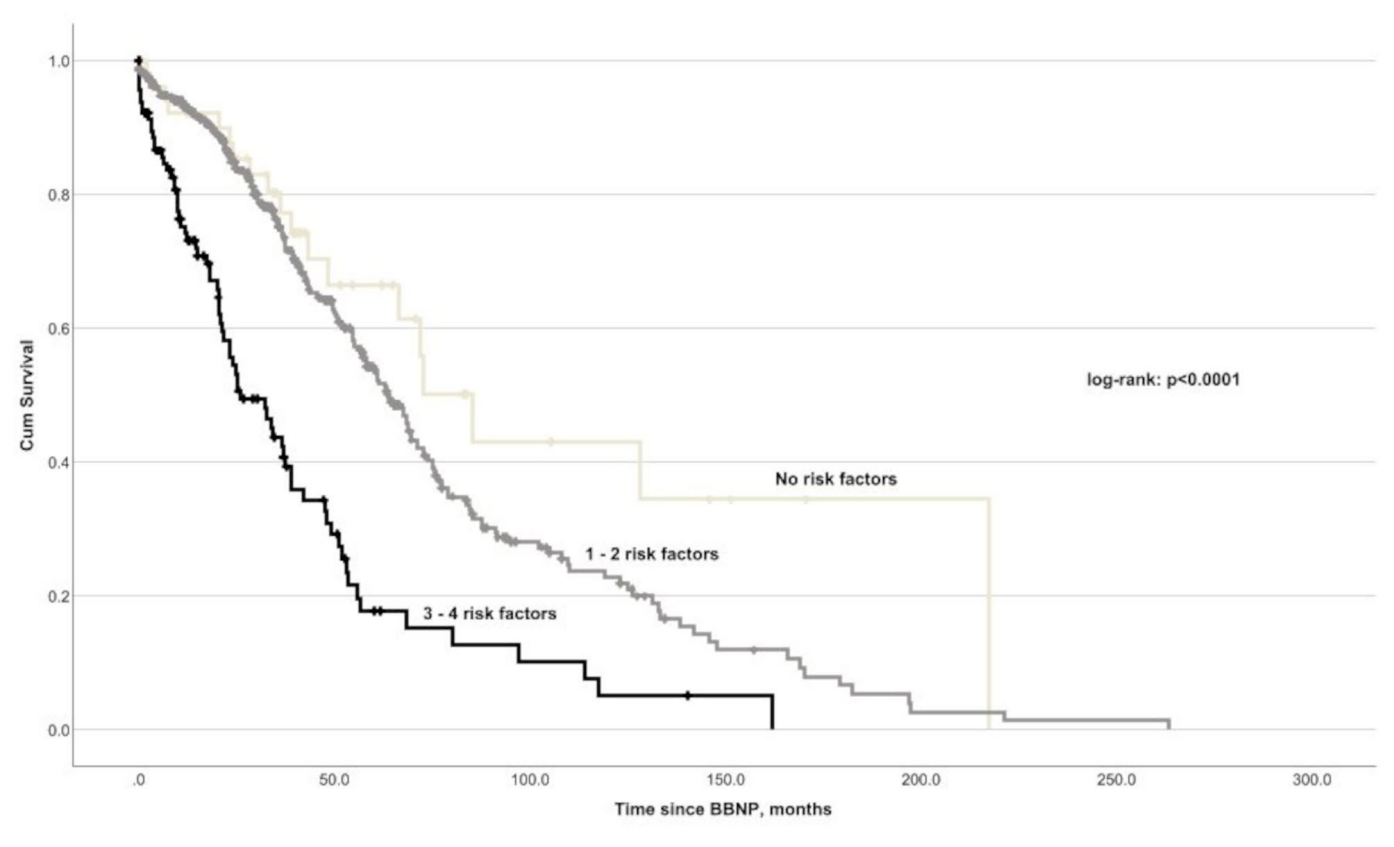
Kaplan-Meier-survival plot showing different timing of LB depending on the number of present predictors in the cohort: patients’ age > 67 years, tumor thickness > 8.5 mm, visual acuity at diagnosis > 0.5 LogMAR, and the absence of ciliary body involvement

Using the Kaplan–Meier survival analysis, we showed the cumulative effect of the significant predictors on occurrence and timing of LB after BBNP (Fig. 3).

## Discussion

In the present study, we have analyzed visual outcome after therapy of large UM with tumor thickness exceeding 7 mm and managed with BBNP. We have maintained prognostic factors for development of LB following brachytherapy. Patients’ age > 67 years, tumor thickness > 8.5 mm, reduced VA at diagnosis, and the absence of ciliary body involvement were identified as predictors for deterioration of VA and developing of LB. The preservation of VA was possible in approximately in half of the cases.

At initial presentation, VA of 88.2% of patients in our cohort was better than 1.0 LogMAR, and the median VA was 0.4 LogMAR. In contrast, initial median VA of COMS patients with medium sized UM was 0.2 LogMAR [9]. Better initial VA in COMS patients could be related to smaller tumor size compared to large UM in our cohort (median tumor height and largest basal diameters in COMS were 4.6 and 11.5 mm respectively vs. 8.6 and 15.0 mm in our cohort). Low VA at diagnosis has already been reported as a predictive factor for VA deterioration [14, 19, 20]. Moreover, initial VA can serve also as a prognostic indicator regarding local treatment failure as well as UM-related mortality and overall survival [21–23]. The predictive value of initial VA on above mentioned endpoints could be related to larger dimension of tumors in patients with low initial VA [21]. In line with previous reports, we have confirmed the association of low initial VA > 0.5 LogMAR with VA loss after brachytherapy. Of note, this association was independent of tumor size in the multivariate analysis, so VA might be a general prognostic marker not solely related to tumor characteristics.

A poor visual prognosis is well known after therapy of eyes with large UM [23, 24]. In one analysis of 1131 patients treated with brachytherapy with ^125^I, the eyes with large tumors showed more VA loss compared to small and medium tumors [25]. Pagliara et al. analyzed the post-treatment course of VA and estimated that the greatest loss of vision occurred in the interval between the first and third year after treatment. VA retained up to the third year after treatment, was maintained in further course in most cases [26]. 

The mechanisms of VA deterioration are different: direct radiation damage to macula and the optic disc due to near tumor location, or due to a dose-dependent, local vascular injury and intraocular VEGF overproduction leading to increased vascular permeability, closure and proliferation, which clinically manifest as RR, RM and RO [27]. RR and RM were diagnosed in our cohort in 163 (28.6. %) and 189 (33.2%) patients respectively. The reported incidence of RR in literature ranges from 20 to 74% [4, 7, 8, 19, 28–30]. RM is one of well-known complications of brachytherapy and associated with poor visual outcome [31–33]. Commonly, proper and timely management of different iatrogenic complications following episcleral brachytherapy consisting of intravitreal VEGF inhibitors, triamcinolone treatment, laser photocoagulation or scleral path grafting is crucial to preserve the eye, prevent or delay visual loss [7, 33–35]. Taking into account that subclinical retinal vasculopathy signs can be detected with optical coherence tomography angiography directly after brachytherapy, anti-VEGF therapy can be initiated before the manifestation of a clinically evident vasculopathy with subsequent vision loss [27]. Victor et al. showed, that preventive anti-VEGF injections every four months for a minimum of 24 months can delay the development of RM and RO, whereas the impact of this therapy on final visual outcome was uncertain [27]. In contrast, another study explored visual outcome following plaque radiotherapy and prophylactic intravitreal bevacizumab and demonstrated less evidence of cystoid macular edema, less clinical evidence of RM, RO and better VA outcomes [36]. Considering that increased tumor thickness is associated with a higher risk of developing macular edema, and its onset tends to occur earlier [25], patients with larger tumors may derive greater benefit from prophylactic anti-VEGF therapy. Dalvin et al. introduced a nomogram for VA outcome after ^125^I plaque radiotherapy and prophylactic intravitreal bevacizumab, which predicts VA based on clinical or treatment risk factors [19]. 

There are only a few studies addressing the functional outcomes following brachytherapy for large UMs and it’s worth noting that directly comparing functional outcomes of different studies poses challenges due to substantial variability in patient cohorts regarding tumor characteristics, radiation parameters, and outcome definitions. Shields et al. reported a poor VA (> 1.0 LogMAR) five years after brachytherapy of large UMs with thickness > 8 mm in 57% of cases [4]. Another study showed the median VA at two years after brachytherapy of 1.9 LogMAR (counting fingers to hand movements) [3]. We have estimated a better median VA (1.4 LogMAR) at 30 months in our study. A significant better functional outcome with median VA of 0.48 LogMAR has been demonstrated after brachytherapy of large UMs with ^125^I and prophylactic intravitreal antiVEGF injections [25]. 

Apart from the interest in using intravitreal anti-VEGF agents and steroids for preventing and treating RR and RM, there is another approach aimed at enhancing visual outcomes post-plaque therapy. This involves combining brachytherapy with vitrectomy and silicone oil (1000-cSt and 5000-cSt) [37, 38]. The rationale behind this approach is to reduce radiation exposure from ^125^I to neighboring sensitive structures [39, 40]. MacCanel et al. demonstrated improved final VA in patients with silicone oil tamponade compared to controls in a one-to-one matched case–control study [37, 41]. However, it’s crucial to consider the potential serious complications associated with the vitrectomy procedure when contemplating this method [42]. 

Taking into consideration, that eyes harboring tumors with large thickness representing a high-risk cohort for VA deterioration, prophylactic intravitreal anti-VEGF injections and/ or combining with silicon oil tamponade are indeed promising to enhance functional outcome following brachytherapy.

Previous studies dealing with visual outcome after brachytherapy for UM determined parameters associated with severe VI and blindness. Patients’ age, tumor thickness, shorter distance between the tumor and the foveal avascular zone, applied apex dose, low initial VA, direct macular involvement, posterior tumor extension were identified as independent prognostic factors for poor visual outcome [9, 13, 20, 24, 43–45]. We have identified tumor thickness > 8.5 mm and patients age > 67 years as independent predictors for visual loss. Fittingly, increased tumor height and increased patient age have been already described as most consistent predictors of ocular morbidity and the most significant risk factors for vision loss following brachytherapy [4, 23]. The only protective factor against visual loss following brachytherapy, as identified in our study, was the involvement of the ciliary body. This could be related to peripheral tumor location which offers an advantage to minimize radiation exposure to critical radiosensitive structures, including optic nerve and fovea.

### Study limitations

The retrospective design and heterogeneity of follow up duration are main limitations of our study. Additionally, utilization of a specific bi-nuclide plaque type, which is unique to our institute, limits the generalizability of our findings. Nevertheless, our study is conducted on one of the largest consecutive single-institutional cohorts and presents a comprehensive analysis of different parameters and their predictive role on visual outcome following brachytherapy for large UM.

## Conclusion

In our study, we have evaluated the functional outcomes following BBNP for large UMs. Most of the patients showed deterioration of VA, with LB occurring in 50,4% of the cases. Predicting visual acuity after brachytherapy for large uveal melanomas is challenging, nevertheless we could identify independent predictors for VA deterioration: patients’ age > 67 years, tumor thickness > 8.5 mm, reduced VA at diagnosis, and the absence of ciliary body involvement. While loss of functionality due to radiotherapy is unavoidable for certain tumor locations, preservation of visual acuity in many cases is possible. Further optimization of treatment strategies, incorporating both therapeutic and preventive measures, has the potential to improve functional outcomes following episcleral plaque therapy for large UMs.

## Data Availability

No datasets were generated or analysed during the current study.
